# Combined analysis of estradiol and β-hCG to predict the early pregnancy outcome of FET: a retrospective study

**DOI:** 10.1186/s13048-024-01433-0

**Published:** 2024-06-21

**Authors:** Man Wu, Xiao Xiao, Chen Wang, Min Zhao, Fang Xiong, Xin Jin, Xiaomin Zheng

**Affiliations:** Wuxi Maternity and Child Health Care Hospital, Affiliated Women’s Hospital of Jiangnan University, Wuxi 214002, China

**Keywords:** Estradiol, β-hCG, Embryo transfer, Pregnancy, Assisted reproductive techniques

## Abstract

**Background:**

The accurate prediction of pregnancy outcomes in in vitro fertilization (IVF) cycles is crucial. While several studies have been conducted on the predictive power of serum estradiol (E_2_) and β-hCG concentrations post-embryo transfer (ET) for pregnancy outcomes, there is debate on the predictive value of E_2_. The objective of this study was to investigate the predictive efficacy of combining serum E_2_ and β-hCG levels on early reproductive outcomes 12 days after embryo transfer.

**Methods:**

A total of 1521 patients with β-hCG positive values on day 12 following frozen-thawed embryo transfer (FET) with natural endometrial preparation cycles (NCs) were gathered in affiliated Women’s Hospital of Jiangnan University. Using logistic regression, the relationship between pregnancy outcome and early serum E_2_ and β-hCG concentrations was examined. The receiver-operating characteristic (ROC) analysis was used to assess the predictive accuracy of the serum E_2_ and β-hCG concentrations.

**Results:**

Notable distinctions were observed in the serum E_2_ and β-hCG levels on the twelfth day following FET with NCs between the groups classified as clinical pregnancy group (CP Group) and biochemical pregnancy group (BP Group). In addition, the cutoff values for E_2_ and β-hCG on day 12 following FET with NCs in cleavage embryo group (CE Group) were 129.25 pg/mL and 156.60 mIU/mL, respectively. The threshold values for E_2_ and β-hCG for the blastocyst group (B Group) were 174.45 pg/mL and 217.70 mIU/mL. Serum E_2 day12_ and β-hCG _day12_ were found to be substantially linked with clinical pregnancy by logistic regression analysis.

**Conclusions:**

Serum E_2_ and β-hCG concentrations were found to be significantly different between the CP Group and BP Group in infertility women underwent FET with NCs. Our retrospective cohort study’s findings suggest that the combination of early E_2_ and β-hCG levels on day 12 post-FET could be used as a predictive tool to evaluate the likelihood of both positive and negative pregnancy outcomes in FET with NCs.

## Introduction

In the field of in vitro fertilization (IVF), which has been in development for over forty years, physicians frequently use β-hCG to forecast the likelihood of successful pregnancy outcomes in women who have conceived naturally or with the use of assisted reproductive technology (ART) [1, 2]. However, there remains ongoing debate regarding the most effective methods for predicting adverse pregnancy outcomes, such as biochemical pregnancies, in order to facilitate a smooth transition from clinical pregnancy to term and minimize the occurrence of biochemical pregnancies.

The role of estradiol (E_2_) is well documented in IVF cycles, particularly in fertilization and implantation. Many researches in hints that successful IVF and a healthy pregnancy is associated with the levels of estrogen. Currently, a number of studies suggested that levels of hormones such as E_2_ may play a role in predicting successful pregnancy in IVF cycles [3, 4], but some results are controversial [5, 6]. What’s more, most previous studies paid attention to the association of clinical outcomes with E_2_ on the day of β-hCG administration, or E_2_ related with fresh embryo transplantation. However, rare study investigated earlier serum E_2_ levels on day 12 after frozen-thawed embryo transfer (FET) and lately clinical pregnancy outcomes. Hormonal replacement treatment cycles (HRTs) and natural endometrial preparation cycles (NCs) are two popular protocols for endometrial preparation, and this retrospective study aimed to assess the association between earlier serum E_2_ and β-hCG levels on day 12 following FET with NCs and clinical pregnancy outcome. Furthermore, our study aimed to identify the optimal E_2_ and β-hCG threshold values for predicting pregnancy outcomes in FET with NCs.

## Materials and methods

All procedures in this study were approved by the Ethics Committee of Affiliated Women’s Hospital of Jiangnan University (2023-06-1213-65).

### Cohort selection

In this retrospective study, infertile women undergoing FET with NCs were included based on electronic medical records from 2012 to 2022.

Clinical characteristics such as maternal age at diagnosis, nationality, the years of infertility, body mass index (BMI), basal estradiol (bE_2_), basal follicle-stimulating hormone (bFSH) and basal luteinizing hormone (bLH), anti-Müllerian hormone (AMH), basal prolactin (bPRL), β-hCG levels, etc., were collected. Weight and height at time of diagnosis were measured by BMI calculated and classified according to the ethnicity-specific WHO classification for Asian/Indian women: underweight (< 18.4 kg/m^2^), normal weight (18.5–22.99 kg/m^2^), overweight (23–27.49 kg/m^2^), and obese (> 27.50 kg/m^2^) [7].

The study included the patients aged up to 35 years old and without major system disease. Patients with uterine malformation, uterine fibroids or adenomyosis, severely damaged endometrium and chromosomal abnormalities of either part of the couple were excluded. Patients with incomplete basic endocrine parameters and untimely β-hCG and E_2_ data were also excluded from the study. Additionally, patients with an endometrial thickness less than 8 mm were not included in the analysis. Those who required exogenous estrogen supplements were also excluded due to their slow endometrial growth without supplementation. Finally, a total of 1521 cycles with positive β-hCG values were collected, forming two groups: CE Group (*n* = 595) and B Group (*n* = 926).

**Fig. 1:**
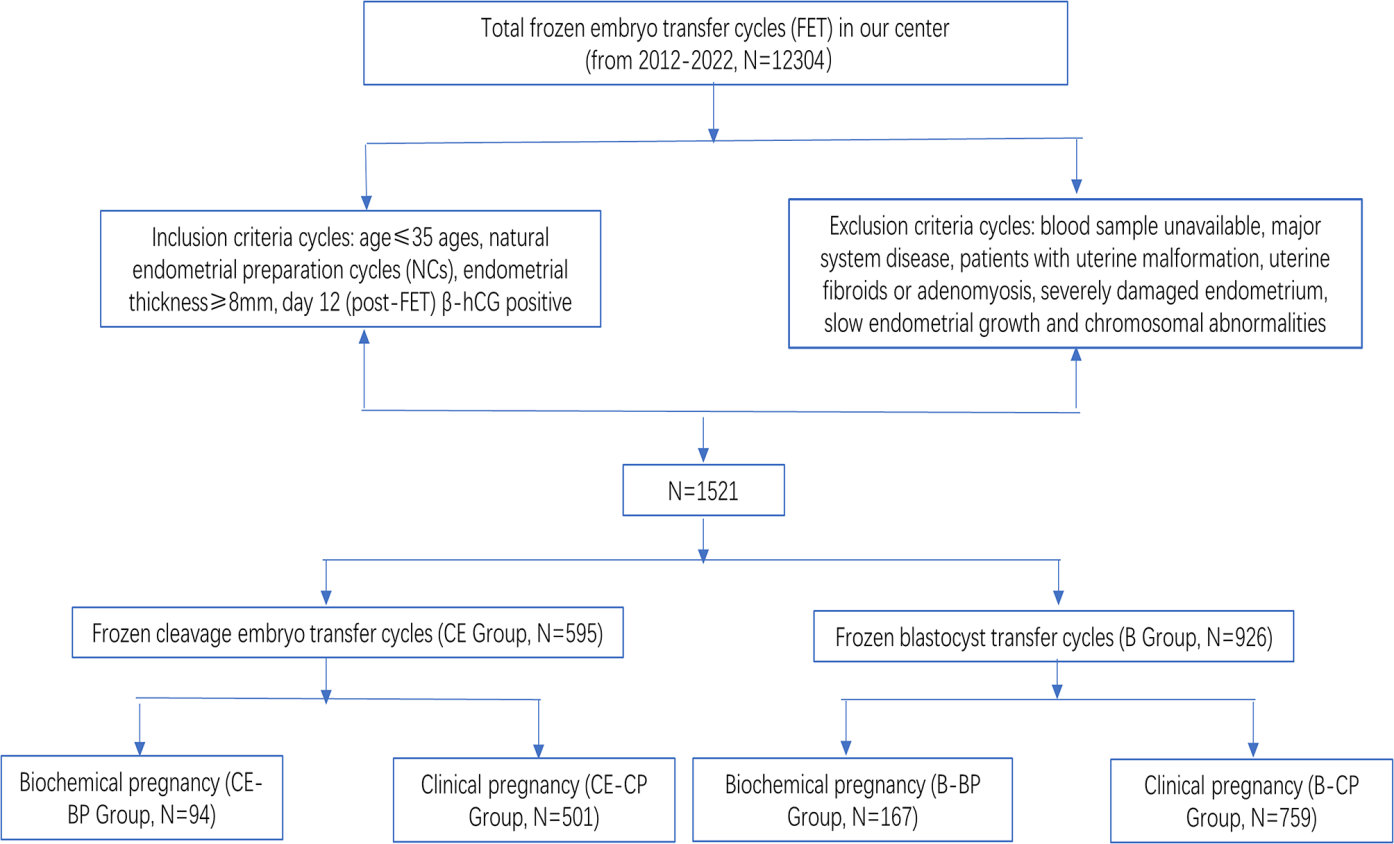
Detail criteria and selection of the cohort selection. FET: frozen-warmed embryo transfer; NCs: natural endometrial preparation cycles; CE: cleavage embryo; B: blastocyst; CE-CP: cleavage embryo to clinical pregnancy; CE-BP: cleavage embryo to biochemical pregnancy; B-CP: blastocyst to clinical pregnancy; B-BP: blastocyst to biochemical pregnancy

Blood samples were collected on day 12 post-embryo transfer, followed by quantification of serum E_2_ and β-hCG levels. The CE Group was further divided into clinical pregnancy group (CE-CP Group, with gestational sac) and biochemical pregnancy group (CE-BP Group, without gestational sac), based on the cutoff value of β-hCG ≥ 5 mIU/mL [8]. Similarly, the B Group was divided into blastocyst-clinical pregnancy group (B-CP Group) and blastocyst-biochemical pregnancy group (B-BP Group). A flow diagram of the patient-selection process is presented in Fig. 1.

### Treatment protocols and endometrial preparation

In the natural FET cycles, endometrial maturation is determined by endogenous estradiol and progesterone, which are generated by stimulating the growth of the endometrium and the development of follicles. Regularly menstruating women can use NCs to plan FET without the need for high dose exogenous hormone supplements. However, oral low-dose estrogen (Estradiol Valerate, Bayer, Germany) will be administered to certain patients whose endometrium thickness is consistently thin, particularly when the dominant follicle is up to 14 mm, in order to promote endometrial growth. Finally, FET will be carried out if the endometrial thickness is ≥ 8 mm. Otherwise, the cycles will be cancelled. Furthermore, for those who might not make enough progesterone during embryo transfer, progesterone treatment is carried out during natural cycles.

Following 12–14 days of menstruation, an ultrasound evaluation was done to track follicle growth and measure follicle size. When the follicle reaches a diameter of 16 mm, daily transvaginal ultrasound becomes necessary, and serum levels of LH, E_2_, and P can be detected until spontaneous ovulation. It is routine to do NCs-FET at the cleavage embryo and blastocyst on Days 0 + 3 and 0 + 5 days, respectively, taking into account that the day of ovulation is Day 0. For luteal phase support, oral progesterone tablets (Duphaston, Abbott, 20 mg) and vaginal progesterone gels (Crinone, Merck Serono, Germany, 90 mg qd) were used. Support for the luteal phase was continued until week 10, at when the β-hCG tests turned positive.

### Morphological assessment of cleavage embryos and blastocysts

D_3_ cleavage embryos were divided into four groups based on their cell number, symmetry of blastomeres, and degree of fragmentation. Grade I included embryos with 7–9 cells, symmetrical blastomeres, and < 5% fragmentation; Grade II included embryos with 6 cells, symmetrical blastomeres, and < 5% fragmentation; Grade III included embryos with 4–5 cells, symmetrical blastomeres, and < 5% fragmentation; Grade IV included embryos with < 4 cells. We classified Grade I and Grade II as high-quality embryos.

Blastocyst morphology was assessed based on the Inner cell mass (ICM) and trophectoderm (TE) which were categorized according to the number and structure of cells. The appearance of tightly packed ICM/TE cells was defined as “A”; several grouped cells as “B”; a few loose cells as “C”. High-grade blastocysts were defined as AA, AB, BA or BB expanded or hatched blastocysts with high ICM/TE grading.

### Hormone assay

The levels of serum E_2_ and β-hCG were quantified using commercially available automated electrochemiluminescence immunoassays (DxI 800 Immunoassay System, Beckman Coulter, USA). All measurements were performed by skilled technicians in strict adherence to the manufacturer’s instructions.

### Outcomes

Pregnancy outcomes were as follows: biochemical pregnancy, clinical pregnancy, ongoing pregnancy, and live birth [9]. Biochemical pregnancy [10] was defined as a serum β-hCG level ≥ 5 mIU/mL 12 days after embryo transfer, and that fails to progress to the point of ultrasound confirmation of gestational sac 28–35 days after embryo transfer. Clinical pregnancy was defined as an intrauterine/extra uterine gestational sac that was detected 28–35 days after embryo transfer by ultrasound.

### Statistical analysis

IBM SPSS Statistics for Windows Version 27.0 (Armonk, NY: IBM Corp) software was used to analyze all the data. For analysis, we performed Kolmogorov-Smirnov test to determine whether variables were normally distributed or not, and then parametric continuous variables were expressed as mean values and standard deviation (¯x ± s), the difference was assessed using T-test. Where required, non-parametric variables were analyzed with the median (1st and 3rd quartiles) and compared using the Mann Whitney U test. A Chi-squared test was used to compare rates.

The receiver-operating characteristic (ROC) analysis was used to assess the predictive accuracy of the serum E_2_ and β-hCG concentrations. AUC measures the diagnostic accuracy of the test, with a greater area corresponding to a greater ability to discriminate between patients with clinical pregnancy or not. AUC < 0.5 indicated no predictive value, 0.5 ≤ AUC < 0.7 indicated a low predictive value, 0.7 ≤ AUC < 0.9 indicated a moderate prediction value and 0.9 ≤ AUC < 1 indicated a high predictive value.

Spearman’s rank correlation (*r*_*s*_) was used to examine the relationship between pregnancy outcome and early serum E_2_ and β-hCG levels. The Pearson’s test was used to assess the difference.

Logistic regression analyses were used to study the association between variables (day 12 serum E_2_ and β-hCG concentration) and pregnancy outcome. Hosmer-Lemeshow test was done to assess the model’s goodness-of-fit.

Statistical significance was set at a two-sided *P* < 0.05.

## Results

### General characteristics of the included patients

**Table 1:** General characteristics of the included patients

Characteristic	Group (*n* = 1521)							
	CE Group (*n* = 595)				B Group (*n* = 926)			
	CE-CP Group (*n* = 501)	CE-BP Group (*n* = 94)	t/Z/χ2 ^b^	*P* ^b^	B-CP Group (*n* = 759)	B-BP Group (*n* = 167)	t/Z/χ2 ^c^	*P* ^c^
Age (¯x ± s) ^a^	30(28, 32)	31(28, 33)	1.181	0.237	30(28, 32)	31(28, 33)	1.766	0.077
Duration of infertility ^a^ (years, ¯x ± s)	3.17 (2.0, 5.0)	3.875 (2.0, 6.52)	1.712	0.087	3.83 (2.58, 5.67)	3.67 (2.42, 5.75)	-0.226	0.821
BMI (¯x ± s, kg/m^2^)	21.63 ± 2.73	21.07 ± 2.50	-1.857	0.064	21.71 ± 2.91	21.46 ± 2.72	-1.007	0.314
bFSH (mIU/mL)	7.45 ± 2.06	7.01 ± 2.03	-1.918	0.056	7.22 ± 2.26	7.29 ± 2.12	0.352	0.725
bE_2_ (pg/mL)	36.33 ± 26.86	41.18 ± 17.81	1.684	0.093	38.20 ± 18.10	40.31 ± 21.19	1.320	0.187
bPRL (ng/mL)	15.32 ± 7.68	15.29 ± 7.33	-0.036	0.971	15.11 ± 6.26	14.93 ± 6.85	-0.331	0.740
bLH (mIU/mL)	4.82 ± 3.70	4.60 ± 1.85	-0.552	0.581	5.43 ± 4.50	5.13 ± 3.39	-0.812	0.417
AMH (ng/mL)	4.08 ± 2.58	3.68 ± 2.51	-1.387	0.166	4.33 ± 2.71	4.11 ± 2.64	-0.950	0.342

CE-CP: cleavage embryo-clinical pregnancy; CE-BP: cleavage embryo-biochemical pregnancy; B-CP: blastocyst-clinical pregnancy; B-BP: blastocyst-biochemical pregnancy; ^a^ Data are median and 25–75 centile range in parentheses; P ^b^ and t/Z/χ2 ^b^ values for comparison between CE-CP Group and CE-BP Group; P ^c^ and t/Z/χ2 ^c^ values for comparison between B-CP Group and B-BP Group

The general characteristics of the included patients are presented in Table 1. No statistically significant differences were observed between the CE-CP Group and CE-BP Group, as well as within the B-CP Group when compared to the B-BP Group.

### Serum E_2_ and β-hCG were significantly higher in clinical pregnancy

Serum E_2_ levels were compared among all groups on day 12 following FET with NCs revealing significantly higher serum E_2_ levels in the CE-CP Group compared to the CE-BP Group (212.04 ± 151.27 vs. 112.18 ± 43.18 pg/mL, *P* < 0.001). Similarly, there was also a significant difference observed between B-CP Group and B-BP Group (215.06 ± 96.80 vs. 135.87 ± 55.20, *P* < 0.001). Likewise, there was significant difference observed regarding β-hCG level on day 12 following FET with NCs between CE-CP Group versus CE-BP Group (474.73 ± 351.88 vs. 69.18 ± 43.41 mIU/mL, *P* < 0.001) and similarly within B Group (832.88 ± 694.36 vs. 107.49 ± 88.44 mIU/mL, *P* < 0.001).

No significant differences were observed in terms of the average number of embryos transferred, the number of high-quality embryos transferred, and endometrial thickness between B-CP Group and B-BP Group, as well as between CE-CP Group and CE-BP Group (*P* > 0.05, Table 2).

**Table 2:** Cycle parameters and serum E_2_ and β-hCG levels on day 12 following FET with NCs

Cycle parameters	Group (*n* = 1521)								
	CE Group (*n* = 595)				B Group (*n* = 926)				
	CE-CP Group (*n* = 501)	CE-BP Group (*n* = 94)	t/χ2 ^a^	*P* ^a^	B-CP Group (*n* = 759)	B-BP Group (*n* = 167)	t/χ2 ^b^	*P* ^b^	
No. of embryos transferred	1.91 ± 0.52	1.89 ± 0.50	-0.249	0.803	1.53 ± 0.50	1.46 ± 0.50	-1.730	0.084	
No. of high-quality embryos transferred	1.60 ± 0.69	1.53 ± 0.74	-0.854	0.393	1.00 ± 0.68	0.89 ± 0.66	-1.922	0.055	
Endometrial thickness (mm)	10.17 ± 1.71	10.04 ± 1.74	-0.683	0.495	10.25 ± 1.68	10.40 ± 1.81	1.048	0.295	
Day 12 E_2_ levels (pg/mL)	212.04 ± 151.27	112.18 ± 43.18	-12.34	<0.001	215.06 ± 96.80	135.87 ± 55.20	-14.317	<0.001	
Day 12 β-hCG levels (mIU/mL)	474.73 ± 351.88	69.18 ± 43.41	-24.81	<0.001	832.88 ± 694.36	107.49 ± 88.44	-27.78	<0.001	

CE-CP: cleavage embryo-clinical pregnancy; CE-BP: cleavage embryo-biochemical pregnancy; B-CP: blastocyst-clinical pregnancy; B-BP: blastocyst-biochemical pregnancy; P ^a^ and t/χ2 ^a^ value for comparison between CE-CP Group and CE-BP Group; P ^b^ and t/χ2 ^b^ value for comparison between B-CP Group and B-BP Group

### **AUC analysis hormone profile of serum E**_**2**_**and β-hCG levels in predicting clinical pregnancy following FET with NCs**

**Fig. 2:**
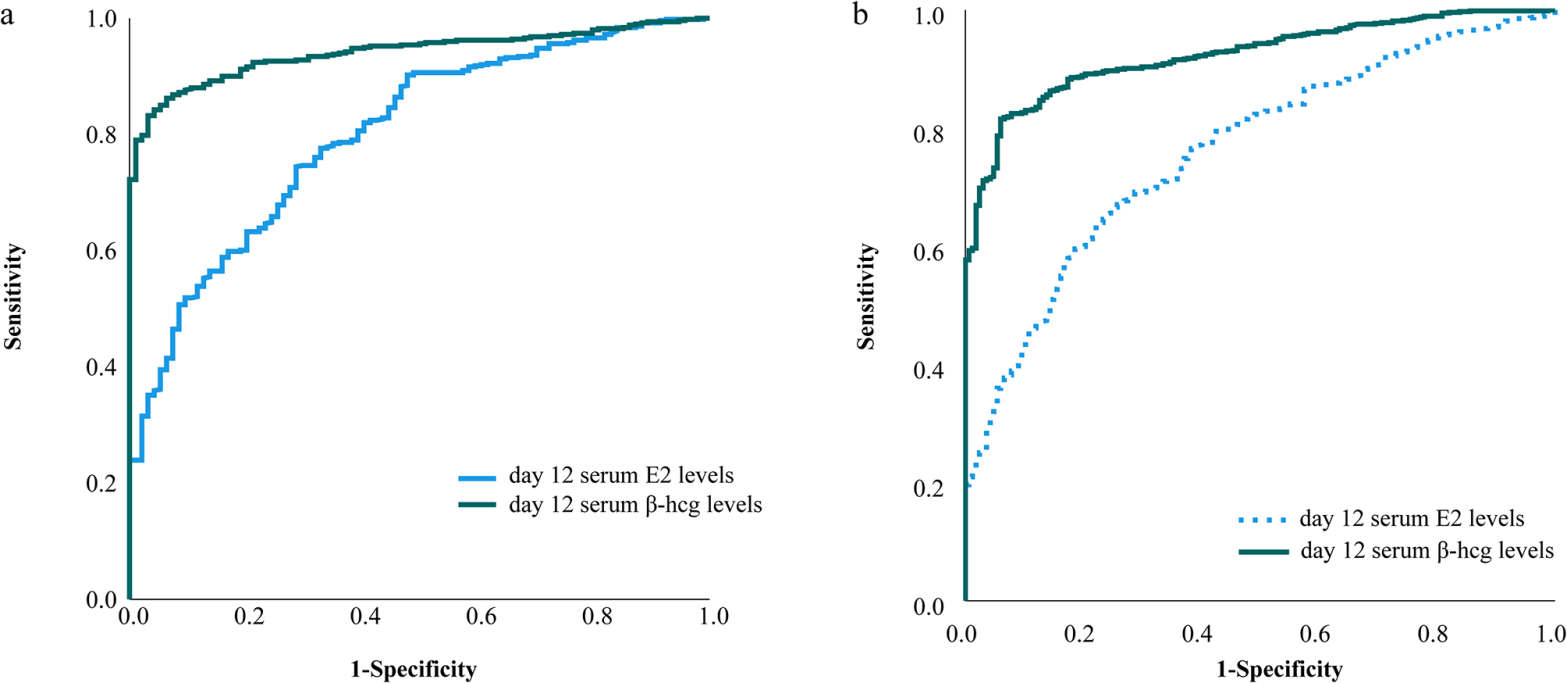
ROC curves for predicting pregnancy outcomes by serum E_2_ and β-hCG levels 12 days following FET with NCs. **(a)** ROC curve analysis of day 12 serum E_2_ and β-hCG levels following FET with NCs in Group CE; **(b)** ROC curve analysis of day 12 serum E_2_ and β-hCG levels following FET with NCs in Group B

The ability of serum E_2_ and β-hCG levels to predict transplant outcomes was evaluated using AUC analysis (Fig. 2). For the CE Group, serum E_2_ levels demonstrated moderate predictive ability for clinical pregnancy (AUC = 0.801; sensitivity = 74.45%; specificity = 71.28%; cutoff value = 129.25 mIU/mL). In terms of β-hCG levels, a cutoff value of 156.60 mIU/mL was identified to predict clinical pregnancy with high sensitivity (84.20%), specificity (95.74%) and predictive value.

For the Group B, the cutoff values for E_2_ and β-hCG concentrations were determined as predictors of clinical pregnancy probability: 174.45 pg/mL for E_2_ and 217.70 mIU/mL for β-hCG respectively. These values yielded AUCs of 0.762 and 0.924 respectively along with sensitivities of 63.77% and 81.56% respectively, specificities of 77.84% and 94.01% respectively. The differences were all significant.

### Significant relations between day 12 E_2_ and β-hCG concentrations

Significant correlations between serum day 12 E_2_ and β-hCG concentrations were found in the CE Group (*r*_*s*_=0.117; *P* = 0.004) and B Group (*r*_*s*_=0.170; *P* < 0.001).

### Association between variables and clinical outcome by logistic analyses

Logistic regression analysis revealed that serum day 12 E_2_ and β-hCG concentrations were the significant predictors of clinical pregnancy (Table 3). The analysis revealed that serum E_2_ and β-hCG on day 12 were significantly associated with clinical pregnancy in the CE Group [E_2 day 12_: odds ratio (OR) of 0.978 (95% CI 0.972–0.984); β-hCG _day 12_: OR of 0.979 (95% CI 0.973–0.984)] and B Group [E_2 day 12_: OR of 0.986 (95% CI 0.983–0.989); β-hCG _day 12_: OR of 0.988 (95% CI 0.986–0.991)].

**Table 3:** Predictive accuracy of serum E_2_ and β-hCG concentrations on day 12 following FET with NCs

	Odds ratio (95% CI)	*p*	AUC (95%CI)	cutoff value	Sensitivity	Specificity
*Prediction of clinical pregnancy of cleavage embryos*						
Day 12 E_2_ levels (pg/mL)	0.978 (0.972–0.984)	*P* < 0.001	0.801 (0.756–0.846)	129.25 mIU/ml	74.45%	71.28%
Day 12 β-hCG levels (mIU/mL)	0.979 (0.973–0.984)	*P* < 0.001	0.941 (0.923–0.959)	156.60 mIU/ml	84.20%	95.74%
*Prediction of clinical pregnancy of blastocyst embryos*						
Day 12 E_2_ levels (pg/mL)	0.986 (0.983–0.989)	*P* < 0.001	0.762 (0.726–0.799)	174.45 mIU/ml	63.77%	77.84%
Day 12 β-hCG levels (mIU/mL)	0.988 (0.986–0.991)	*P* < 0.001	0.924 (0.907–0.941)	217.70 mIU/ml	81.56%	94.01%

CI = confidence interval

## Discussion

In recent years, IVF has successfully addressed various causes of infertility. However, accurately predicting of IVF cycle outcomes remains a critical concern. Previous research has indicated a correlation between β-hCG levels at different time points after fresh embryo transfer or on the day of ovulatory dose of hCG administration along with E_2_ levels and pregnancy outcomes. Nevertheless, the predictive value of E_2_ is still controversial. Therefore, this study aims to assess the predictive value of serum E_2_ and β-hCG concentrations on day 12 in determining early reproductive outcomes for women undergoing FET with natural cycles using natural cycles. We observed significant differences in serum E_2_ levels between biochemical pregnancy group and clinical pregnancy group, and proposed cutoff values for both E2and β-hCG. Moreover, a significant correlation between serum E_2_ and β-hCG was found in CE Group and B Group.

Previous research has suggested that E_2_ levels during the luteal phase may serve as a predictive factor for ongoing pregnancy post-clinical pregnancy [4, 11, 12]. However, conflicting results have been reported [5, 6]. Goldman RH et al. [5] found that elevated serum E_2_ levels at the initiation of progesterone treatment may adversely affect implantation, pregnancy, and live birth outcomes following FET. Excessive serum E_2_ levels could negatively impact the chances of conception and live birth after IVF. Huang’s study [13] demonstrated that E_2_ influence during the peri-implantation period (day 11) does not affect clinical outcomes in fresh embryo transplantation. A meta-analysis by Karatasiou GI et al. [14] revealed insufficient evidence to support or refute an association between serum E_2_ levels on the day of triggering final oocyte maturation with hCG and the probability of pregnancy. A study using mouse models showed that variations in E_2_ levels play an crucial role in determining the window of opportunity for embryo attachment to the uterine wall and subsequent development [15]. Additionally, another study indicated a potential positive correlation between higher E_2_ levels and improved pregnancy outcomes [16]. Furthermore, recent research has linked decreased E_2_ levels on cycle day 28 (22 days post fresh embryo transfer) to poorer pregnancy outcomes [17]. There are some methodological differences as well as variations in patient populations between these studies and our research. Our focus is specifically on infertile women who underwent FET with NCs, aiming to evaluate the association between E_2_ level and clinical outcomes.

Current clinical methods for predicting pregnancy outcomes often utilize a combination of β-hCG and P levels, particularly in women undergoing ART or conceiving naturally [1, 18]. Normally, serum β-hCG and P are lower in nonpregnant women, and their levels could rise significantly after pregnancy. Previous studies have demonstrated that β-hCG levels at 7 or 14 days after transplantation can be used as predictors of pregnancy outcomes [19, 20]. Additionally, serum β-hCG levels on postovulatory day 12 and 14 can also serve as predictors of pregnancy outcome following an IVF-ET cycle [21]. However, the association between serum E_2_ and β-hCG levels on day 12 following FET with natural cycles has not been reported in previous studies. This gap is addressed by our research.

In contrast to current clinical methods for predicting pregnancy outcomes, our study distinguishes itself in several key ways. Firstly, we specifically focus on FET cycles to eliminate the confounding effects of high-dose hormones typically associated with fresh embryo transfer during ovulation induction cycles. Secondly, we concentrate on endometrial preparation for transfer regimens in natural cycles, thereby excluding the influence of exogenous hormones on predicted outcomes. Lastly, our study focuses on FET with natural cycles and proposed the cutoff values of E_2_ (129.25 pg/mL for CE Group and 174.45 pg/mL for B Group) for predicting clinical pregnancy following FET with NCs, which is a relatively new perspective on predicting pregnancy outcomes.

Uterine decidualization supports embryo implantation and placentation, as well as subsequent events that maintain a successful pregnancy together [22]. Recent research suggests that endometrial receptivity rather than embryo quality is a key factor restricting successful pregnancy after FET. HRTs or NCs which were usually used for the endometrial preparation [23] can be observed as an important factor affecting the pregnancy rate of FET. In order to eliminate the impact of hormone supplementation, our study focuses on infertile women who underwent FET with natural cycles and aims to evaluate the association between E_2_ levels and clinical outcomes.

To date, limited research has explored the relationship between pregnancy outcomes and E_2_ levels on day 12 following FET with NCs. In this retrospective cohort study, we examined the correlation between E_2_ levels and clinical outcomes in infertile women undergoing FET with NCs. Additionally, we established E_2_ cutoff values that, when combined with β-hCG levels on day 12 following FET with NCs, can be used to assess both positive and negative pregnancy outcomes. Our findings indicate that E_2_ levels were significantly higher in the clinical pregnancy group compared to the biochemical pregnancy group on day 12 following FET with NCs, suggesting a potential correlation between E_2_ levels and clinical outcomes. Lower E_2_ levels may potentially affect endometrial gland development and maturation, leading to a decrease in clinical pregnancy rates. By evaluating serum E_2_ levels alongside β-hCG levels on day 12 post-FET, healthcare providers can enhance outcome predictions and customize treatment plans for patients. This approach may also lead to a reduction in patient visits and clinical serum hormone tests, ultimately decreasing psychological stress.

Although our study effectively establishes a correlation between E_2_ levels and clinical pregnancy outcomes in infertile women undergoing FET with NCs, there are deficiencies in our research. There are limitations inherent in its retrospective design which weaken its statistical power. Additionally, the specific thresholds for elevated E_2_ levels affecting endometrial receptivity remain uncertain.

## Conclusion

Our findings suggest that there is a significant difference in serum E_2_ levels between biochemical and clinical pregnancies following FET with NCs, and we have proposed E_2_ cutoff values for predicting clinical pregnancy in this context. The results of our retrospective cohort study indicate that the cutoff value of E_2_ for predicting clinical pregnancy with acceptable sensitivity and specificity is 129.25 pg/mL for CE Group and 174.45 pg/mL for B Group.

In conclusion, incorporating both early E_2_ and β-hCG levels on day 12 post-FET may serve as a predictive tool for assessing both positive and adverse pregnancy outcomes in FET with natural cycles. These findings suggest that in addition to serum β-hCG concentrations on day 12, serum E_2_ levels on the same day can also be indicative of clinical pregnancy following FET with NCs. Furthermore, both serum E_2_ and β-hCG levels on day 12 are equally valuable in predicting clinical pregnancies, showing a significant correlation between them. Therefore, integrating day 12 E_2_ levels into routine follow-up protocols may be beneficial.

## Data Availability

No datasets were generated or analysed during the current study.

## Abbreviations

FET: Frozen-thawed embryo transfer
NCs: In natural endometrial preparation cycles
CP Group: Clinical pregnancy group
BP Group: Biochemical pregnancy group
CE Group: Cleavage embryo group
B Group: Blastocyst group
IVF-ET: In vitro fertilization-embryo transfer
ART: Assisted reproductive techniques
E_2_: Estradiol
β-hCG: β-human chorionic gonadotropin
P: Progesterone
HRTs: Hormonal replacement treatment cycles
ROC: Receiver operating characteristic
AUC: Area under the ROC curve
CI: Confidence interval
